# Lupine embryo axes under salinity stress. II. Mitochondrial proteome response

**DOI:** 10.1007/s11738-013-1273-2

**Published:** 2013-04-03

**Authors:** Łukasz Wojtyla, Arkadiusz Kosmala, Małgorzata Garnczarska

**Affiliations:** 1 Department of Plant Physiology, Faculty of Biology, Adam Mickiewicz University, ul. Umultowska 89, 61-614 Poznań, Poland; 2 Laboratory of Cytogenetics, Institute of Plant Genetics, Polish Academy of Sciences, ul. Strzeszyńska 34, 60-479 Poznań, Poland

**Keywords:** Salinity stress, Mitochondria, Protein

## Abstract

Germination is the first step of plant growth in plant life cycle. An embryonic radicle protruding the seed coat is the first part of plant which has direct contact with external environment including salt-affected soil. In embryo axes, mitochondria are the main energy producer. To understand better salinity impact on mitochondria functioning, this study was focused on the effect of NaCl stress onto mitochondria proteome. Mitochondria were isolated from yellow lupine (*Lupine luteus* L. ‘Mister’) embryo axes cultured in vitro for 12 h with 250 and 500 mM NaCl. Two-dimensional gel electrophoresis of mitochondrial proteins isolated from NaCl-treated axes demonstrated significant changes in proteins abundances as a response to salinity treatment. Twenty-one spots showing significant changes in protein expression profiles both under 250 and 500 mM NaCl treatment were selected for tandem mass spectrometry identification. This approach revealed proteins associated with different metabolic processes that represent enzymes of tricarboxylic acid cycle, mitochondrial electron transport chain, enzymes and proteins involved in mitochondria biogenesis and stresses response. Among proteins involved in mitochondria biogenesis, mitochondrial import inner membrane translocase, subunit Tim17/22, mitochondrial-processing peptidase subunit alpha-1, mitochondrial elongation factor Tu and chaperonins CPN60 were revealed. Finally, formate dehydrogenase 1 was found to accumulate in lupine embryo axes mitochondria under salinity. The functions of identified proteins are discussed in relation to salinity stress response, including salinity-induced PCD.

**Electronic supplementary material:**

The online version of this article (doi:10.1007/s11738-013-1273-2) contains supplementary material, which is available to authorized users.

## Introduction

Salinity is one of the most widespread abiotic stresses, which has a serious effect on plants growth and breeding. Salt-contaminated soils constitute nearly 10 % of the land surface. This poses a problem mainly for irrigated lands, of which about 50 % are salt-affected (Ruan et al. 2010). The rank of this problem is that although irrigated land accounts for only 15 % of total cultivated land, it produces one-third of the world’s food (Munns and Tester 2008). In Europe, salt affects about 1–3 million ha of soil, mainly in the Caspian Basin, Iberian Peninsula and Mediterranean countries (Ruan et al. 2010).

Changes in metabolism under the influence of stress conditions include almost all the processes and pathways within the cells (Shulaev et al. 2008). The proper course of metabolic processes requires sufficient amount of biologically useful energy, usually in the form of high-energy phosphate bonds between residues in the ATP. ATP is synthesized in plants in thylakoid membrane and inner mitochondrial membrane by ATP synthase. In cells of the root tissues, mitochondria are the main cellular compartment responsible for ATP production. The functioning of mitochondria and mitochondrial electron transport chain affects the energy balance of the root cells; the reduction in a respiratory activity and oxidative phosphorylation in mitochondria lead to a deterioration in the energy balance of the plant (Jacoby et al. 2011). Moreover, it was proposed that mitochondria may be an important point in mediating responses to a variety of stresses, are also involved in cell adaptation and play a central role in activation of programmed cell death (PCD) (de Pinto et al. 2012).

Salinity stress disturbs homeostasis of Na^+^, K^+^ and Ca^2+^ ion pools in both the cytoplasm and mitochondria and affects activity of mitochondrial enzymes. Osmotic stress leads to increase in the degree of reduction of cell components, resulting in increased production of reactive oxygen species (ROS) in the mitochondrial electron transport chain. Elevated level of ROS and activation of antioxidant systems were observed under salinity stress (Miller et al. 2010). Mitochondrion is the cellular compartment, which is particularly vulnerable to the adverse effects of ROS. Mitochondrial proteins are, at a much greater extent than chloroplast and cytosolic proteins, modified and oxidatively damaged (Sweetlove et al. 2002; Møller and Sweetlove 2010). The most susceptible to oxidative damage are proteins containing iron–sulphur centres, and thiol groups (Navrot et al. 2007). The increase in ROS levels and ionic disturbance lead to an increase in mitochondrial membrane permeability and efflux of cytochrome *c* into the cytosol that results in execution of programmed cell death (PCD) (de Pinto et al. 2012).

There are still only a few literature data indicating participation of mitochondria in plant response to salinity and the role of mitochondrial proteins in stress response. Most studies on the effects of salinity on mitochondria were carried out on the green photosynthetically active tissues, in which chloroplasts participate in ATP production and also are the main source of ROS.

The aim of the present study was to provide new insights into mitochondria response to salinity stress using a proteomic approaches. The experiments were conducted on embryonic axes isolated from lupine seeds and grown in vitro on medium supplemented with NaCl. Two concentrations of salt: 250 and 500 mM were applied. Our previous study focused on ultrastructural characterization of lupine embryo axes cells under salinity stress. Ultrastructural observations and analysis of DNA degradation confirmed the activation of PCD reaction in lupine embryo axes as a response to high salt concentration (Wojtyla et al. 2013). According to the current knowledge of the exposure of plants to salinity, high salt concentration leads to salt shock. Salt shock is defined as an extreme form of salt stress, where plants are exposed suddenly to high level of salinity (Shavrukov 2013). The main component of salt shock is osmotic shock or plasmolysis, especially in root cells (Munns 2002). However, in our experiments, we did not observe plasmolysis in embryo axes cells after 12 h treatment with both salt concentrations (Wojtyla et al. 2013). After NaCl application, plants usually achieve osmotic homeostasis during several hours (Munns 2002). Plants exposed to severe salt shock can experience cell death (Shavrukov 2013). Apoptosis-like cell death was reported in barley (Katsuhara 1997) and rice roots (Chen et al. 2009b), *Thellungiella halophile* suspension-cultured cells (Wang et al. 2010) and in the unicellular green alga *Micrasterias denticulata* (Affenzeller et al. 2009) under salt shock. The identification of salinity stress-responsive proteins localized in plant mitochondria, performed in this study, should shed light into the functioning of mitochondria under salinity, including salinity-induced PCD.

## Materials and methods

### In vitro culture of embryonic axes

Seeds of yellow lupine (*Lupinus luteus* L. ‘Mister’) were obtained from Poznań Plant Breeders Ltd. Seeds were surface-sterilized in 0.02 % mercuric chloride for 5 min and in 0.5 % sodium hypochlorite for 10 min, rinsed and allowed to imbibe for 24 h at 25 °C. Embryonic axes isolated from imbibed seeds were grown in vitro for 12 h on Heller (1953) medium supplemented with 60 mM sucrose (Borek et al. 2006) and with addition of 250 or 500 mM sodium chloride or without NaCl (control). 40 embryo axes were grown in one flask. In vitro culture was carried out in dark at 25 °C on a gyratory shaker (150 rpm).

### Mitochondria isolation

Mitochondrial fraction was obtained by differential centrifugation and purification of mitochondria by centrifugation in Percoll (Sigma) gradient (Małecka et al. 2001). Mitochondria were isolated from 240 embryo axes cultured in 6 flasks for control and each NaCl concentration. The plant material was homogenized in the isolation buffer containing 5 % BSA, 1 mM EDTA, 1 % PVP, 0.35 M sucrose, and 0.05 M KH_2_PO_4_/K_2_HPO_4_ buffer (pH 7.5). The homogenate was centrifuged for 10 min at 3,000×*g*. Then, the supernatant was centrifuged for 20 min at 15,000×*g*. The obtained supernatant was the cytosol fraction. The pellet containing mitochondria and peroxisomes was gently resuspended in a medium consisting of 0.3 M mannitol, 0.2 % BSA, 1 mM EDTA, and 20 mM Mops (pH 7.2) and then purified in a continuous gradient formed by 24 % (v/v) Percoll in 0.25 M sucrose, 0.2 % BSA, and 20 mM Mops (pH 7.2). The gradient was centrifuged at 40,000×*g* for 40 min in fixed angle rotor with vacuum. Afterwards, the mitochondrial fractions were carefully collected and separeted from Percoll and BSA, by washing twice in a buffer (0.35 M succrose, 20 mM Mops, pH 7.2). After 30 min centrifugation at 8,000×*g*, purified mitochondria were resuspended in the same buffer and concentration of protein was estimated according to Bradford method (1976). Mitochondria were used for respiratory measurement and 2D-IEF-PAGE electrophoresis. The integrity and purity of mitochondrial fractions were confirmed by ultrastructure observation in transmission electron microscope.

### Respiratory measurement

Oxygen consumption was measured in a Clark-type oxygen electrode in 0.8 mL of reaction medium containing 0.05 M K_2_HPO_4_/KH_2_PO_4_ buffer, pH 7.5, 0.3 M mannitol, 10 mM KCl, 5 mM MgCl_2_ and 0.2 % BSA. Respiration rate were measured in the presence of 10 mM succinate with 0.5 mg of mitochondrial protein as described by Małecka et al. (2001). Three independent experiments were done; the statistical deviation of mean values was calculated using Student’s *t* test.

### 2D-IEF-PAGE electrophoresis technique

Proteins of mitochondrial fraction were precipitated in 10 % trichloroacetic acid (TCA) in acetone at −20 °C overnight followed by three subsequent washing steps in cold acetone and centrifugation (Magni et al. 2007). The protein pellet was dried and dissolved in 200 μl buffer composed of 7 M urea, 2 M thiourea, 2 % CHES, and 65 mM DTT (Mechin et al. 2003). Protein concentration was measured using modified Bradford (1976) method. The modification was performed using the above-mentioned buffer for the quantification of protein and preparation of standard curve to eliminate interference effect. Isoelectrofocusing of proteins was performed on gel strips of 7 cm length and immobilized pH gradient in the range of 4–7 (Immobilon Dry Strip, GE Healthcare). In order to rehydrate, gel strips were placed in a rehydration buffer containing 7 M urea, 2 M thiourea, 2 % CHES, 65 mM DTT, 100 g of protein, 1 % IPG buffer 4–7 (GE Healthcare), and 0.002 % bromophenol blue (BPB). Rehydration was performed overnight at room temperature. Isoelectrofocusing was performed with Muliphore II from GE Healthcare according to the manufacturer’s manual. After isoelectrofocusing, gel strips were placed for 15 min in 50 mM Tris–HCl buffer pH 8.8 containing 6 M urea, 30 % glycerol, 2 % SDS, 0.002 % BPB, 65 mM DTT, and for the next 15 min in 50 mM Tris–HCl buffer pH 8.8 containing 6 M urea, 30 % glycerol, 2 % SDS, 0.002 % BPB, 2.5 % iodoacetamide, for pH adjustment and protein reduction. Separation of proteins according to their molecular masses was done using denaturing electrophoresis according to Laemmli (1970). After electrophoresis, gels were stained with Coomassie Brilliant Blue G-250 according to the protocol of Candiano et al. (2004) and scanned.

### Protein selection

Analysis of images of 2D-gel was processed with ImageMaster 2-D Platinum GE Healthcare. This processing involved spot detection, normalization, spot matching, expression, and statistical analysis. Protein spots were automatically selected using the parameters (smooth: 2, a minimum area of 5, silency: 1.00000) followed by manual correction of protein spots and matching of protein spots in order to determine the spots corresponding to each other. Comparative analysis was then performed within the repetitions for each variant to determine the average values of vol%. These values were compared with each other within variants. Protein spots showing significant percentage changes higher than 20 % under both NaCl concentrations and reproducible present in all gels were considered to analysis. Selection of the proteins was done according to technical ability to pick each spot separately. Protein spots were picked form gels in sterile condition and send for LC–MS-MS/MS (liquid chromatography coupled to tandem mass spectrometry) identification to Laboratory of Mass Spectrometry of the Institute of Biochemistry and Biophysics Polish Academy of Science Warsaw, Poland.

### Protein identification

Peptide mixtures were analyzed by LC–MS–MS/MS (liquid chromatography coupled to tandem mass spectrometry) using Nano-Acquity (Waters) LC system and Orbitrap Velos mass spectrometer (Thermo Electron Corp., San Jose, CA). Prior to the analysis, proteins were subjected to standard “in-solution digestion” procedure during which proteins were reduced with 100 mM DTT (for 30 min at 56° C), alkylated with 0.5 M iodoacetamide (45 min in darkroom at room temperature) and digested overnight with trypsin (sequencing Grade Modified Trypsin-Promega V5111). Peptide mixture was applied to RP-18 precolumn (nanoACQUITY Symmetry^®^ C18-Waters 186003514) using water containing 0.1 % TFA as mobile phase and then transferred to nano-HPLC RP-18 column (nanoACQUITY BEH C18-Waters 186003545) using an acetonitrile gradient (0–60 % AcN in 120 min) in the presence of 0.05 % formic acid with the flow rate of 150 nL/min. Column outlet was directly coupled to the ion source of the spectrometer working in the regime of data-dependent MS to MS/MS switch. A blank run ensuring lack of cross-contamination from previous samples preceded each analysis. Acquired raw data were processed by Mascot Distiller followed by Mascot Search (Matrix Science, London, UK, on-site license) against FlyBase database. Search parameters for precursor and product ions mass tolerance were 20 ppm and 0.6 Da, respectively, with search parameters set as follows: one missed semiTrypsin cleavage site allowed, fixed modification of cysteine by carbamidomethylation and variable modification of lysine carbamidomethylation and methionine oxidation. Only peptides with Mascot score exceeding the threshold value corresponding to <5 % false positive rate, calculated by Mascot procedure, were considered to be positively identified. Finally, the proteins were identified as the entries with the best Mascot score and with the best similarity between the experimentally and theoretically calculated values of pI and molecular weight.

### Transmission electron microscope

Preparation of mitochondria fraction for transmission electron microscopy (TEM) was carried on according to modified procedure by Borek et al. (2006). Mitochondria pellet was fixed in Karnowsky half-strength fixative (Karnowsky 1965), i.e. in a mixture of 4 % glutaraldehyde and 4 % paraformaldehyde at the ratio 1:1 with 0.3 M sucrose. Post-fixation was conducted in 1 % OsO_4_ with 0.3 M sucrose. The samples were stained in 2 % aqueous solution of uranyl acetate with 0.3 M sucrose. Dehydration was performed in a series of acetone and ethanol solutions. The mitochondria pellet was embedded in epoxy resin of low viscosity (Spurr 1969). Ultrathin sections were prepared using Ultrotome III (LKB), stained in 5 % uranyl acetate and 0.5 % lead citrate and observed under the transmission electron microscope TEM-1200 Ex JEOL.

## Results

### Respiratory measurements and mitochondria observation

**Fig. 1:**
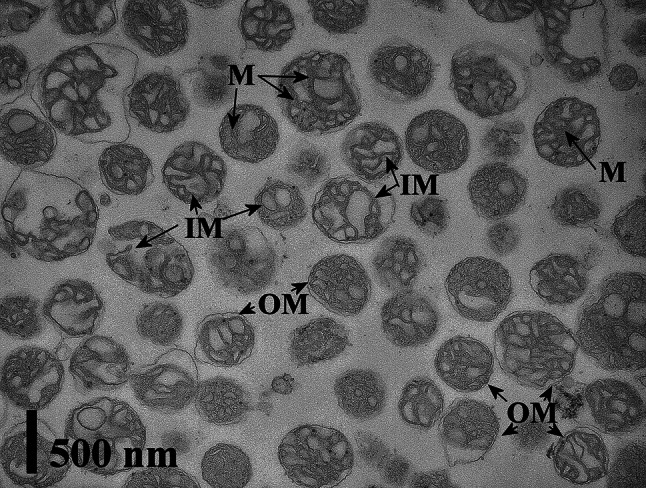
Representative transmission electron micrograph of Percoll-purified mitochondria. The quality and purity of mitochondria isolated from yellow lupine embryo axes treated with 250 mM NaCl was analyzed using transmission electron microscope. In the images, *OM* outer, *IM* inner mitochondrial membrane, and *M* mitochondrial matrix are indicated

Respiratory rate was measured as succinate-dependent oxygen consumption by mitochondria isolated from lupine embryo axes. Respiration rate decreased in relation to NaCl content in growing medium (Table 1). Before the analysis of the mitochondrial respiration, the integrity and purity of mitochondrial fraction from all variants were confirmed by observation of mitochondria pellet in TEM. There were no differences in size and shape of mitochondria. Ultrastructure of isolated mitochondria was very similar in all analyzed variants. Representative electron micrograph of Percoll-purified mitochondria from lupine embryo axes treated with 250 mM NaCl is shown in Fig. 1.

**Table 1:** Mitochondria respiratory rate. Respiration rate of mitochondria isolated from lupine embryo axes grown for 12 h on Heller medium with 250 and 500 mM NaCl or without NaCl

NaCl concentration in the growing medium	0 mM	250 mM	500 mM
Respiration rate ± SD	76.84	34.47	25.33
(nM O_2_ min^−1^ mg of mitochondrial protein^−1^)	±10.91**	±4.03**	±4.02**

** Results differ statistically with *P* < 0.05

### 2-D analysis of salinity-responsive proteins

**Fig. 2:**
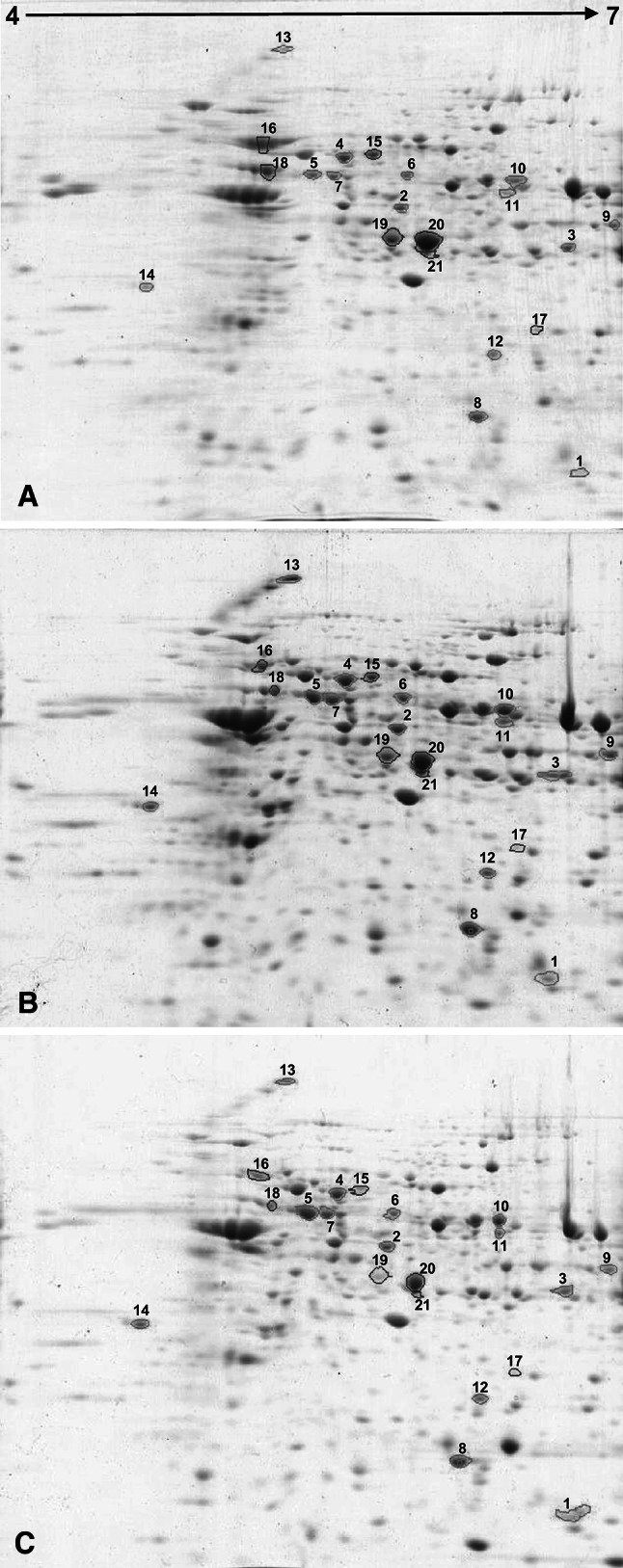
2D-electrophoresis gels. Electrophoresis was conducted in linear pH gradient 4–7 and according to protein molecular masses. Reference gel was obtained by comparison of 2 individual repetition of mitochondrial proteins from each treatment. Samples of mitochondrial proteins were obtained from 10 individual cultures of embryo axes (40 embryo axes each) for control (**a**), 250 (**b**) and 500 (**c**) mM NaCl treatment. 100 μg of mitochondrial protein was loaded on each gels. 21 proteins were chosen for identification, 14 up-regulated proteins are *circled green* and 7 down-regulated are marked with *red circles*

Mitochondrial proteins were extracted from purified mitochondria isolated from embryonic axes treated for 12 h with 250 and 500 mM NaCl as well as from control axes and separated in a first dimension in the linear pH gradient 4–7 (Fig. 2). The protein maps produced from two biologically independent replicates for each experimental variant showed a high level of reproducibility. Analysis of the 2D gels using ImageMaster 2-D Platinum software (GE Healthcare), followed by visual confirmation, revealed the existence of a number of spots showing quantitative differences between treatments. Digital analysis of mitochondrial proteins separation images revealed the presence of 318 proteins spots that could be matched in all of the gels. The intensity of 182 (57 %) and 231 (73 %) spots changed by at least 20 % (change in normalized spot volume, increase or decrease) in the 250 and 500 mM NaCl treatments, respectively, when compared to the control. From these protein spots, 137 showed changes in proteins abundance in both variants. From all 182 significantly changed protein spots revealed after treatment with 250 mM NaCl, 86 (27 %) spots showed increase in the relative intensity of CBB staining, while 96 (30 %) decrease in relation to control gel. After 500 mM NaCl treatment, the relative intensity of staining of 110 (35 %) and 121 (38 %) spots increased or decreased significantly, compared to the control level.

### Protein identification

Twenty-one spots showing significant changes in protein expression profiles both under 250 and 500 mM NaCl treatment and with the best visible spot separation in gels were selected for identification. The identities of these proteins were determined by liquid chromatography coupled with tandem mass spectrometer and are listed in Table 2. The sequences of tryptic peptide matched to sequence of identified protein are listed in electronic supplementary material (Online Resource 1). Soluble proteins from mitochondria lumen and intermembrane space as well as membrane-associated and integral membrane proteins were present in mitochondrial protein fraction. Among 21 identified proteins, 14 were up- and 7 were down-regulated. Thus, a dihydrolipoyllysine-residue acetyltransferase, 24 kDa subunit of NADH-ubiquinone oxidoreductase, ATP synthase subunit α, mitochondrial-processing peptidase subunit alpha-1, mitochondrial elongation factor Tu, formate dehydrogenase 1, manganic superoxide dismutase, heat shock protein 70 kDa, chaperonins CPN60, mitochondrial import inner membrane translocas and subunit Tim17/22 were identified as salinity stress-induced proteins. All identified proteins, both up-and down-regulated, correspond to plant mitochondrial proteins. These proteins belong to different functional classes and represent enzymes of citric acid cycle and mitochondrial electron transport chain, proteins involved in mitochondria biogenesis and stress response such as chaperonins and heat shock proteins as well as pro-oxidative/antioxidative enzymes.

**Table 2:** Protein spots MS/MS identification

Spot no.^a^	NCBI accesion no.^b^	Protein homologous^c^	GO annotations^d^	*M*_r_ (theor./exp.)^e^	pI (theor./exp.)^f^	Score^g^	MQ^h^	SC^i^	0.25 M NaCl %^j^	0.5 M NaCl %^k^
1	gi|87240460	Mitochondrial import inner membrane translocase. subunit Tim17/22 [*Medicago truncatula*]	Integral to membrane	19.3/19	6.97/6.8	566	17	6	+253	+628.2
2	gi|29839695	Mitochondrial-processing peptidase subunit alpha-1 [*Arabidopsis thaliana*]	Response to salt stress, electron transport chain, proteolysis	54.4/47.5	5.94/5.9	259	6	6	+33.5	+31.8
3	gi|25090254	Mitochondrial elongation factor Tu [*Arabidopsis thaliana*]	GTP catabolic process, response to cadmium ion	49.4/42	6.25/6.7	157	4	8	+81.1	+47.7
4	gi|7330642	Heat shock protein 70 kDa [*Solanum tuberosum*]	Protein folding, response to stress	73/70	6.37/5.7	1,695	35	22	+34.7	+56.4
5	gi|12644189	Chaperonin CPN60 [*Arabidopsis thaliana*]	Response to heat, protein refolding, mitochondrion organization, response to cadmium ion	57.6/61.2	5.5/5.7	3,151	74	38	+96.7	+247.6
6	gi|2506275	Chaperonin CPN60-1 [*Zea mays*]	Response to heat, protein refolding, response to cadmium ion	61.2/63	5.68/6.0	1,309	28	34	+48.4	+49.9
7	gi|2493646	Chaperonin CPN60-2 [*Zea mays*]	Response to heat, protein refolding, response to cadmium ion	60.9/57.6	5.7/5.6	2,254	46	38	+100.4	+150.7
8	gi|464775	Superoxide dismutase [Mn] [*Hevea brasiliensis*]	Superoxide metabolic process	25.8/21.7	7.1/6.3	271	8	14	+30.5	+48.9
9	gi|109909540	Formate dehydrogenase 1 [*Oryza sativa*]	Response to cadmium ion, response to wounding	41.3/41.5	6.68/6.85	171	4	7	+75.7	+24.9
10	gi|231585	ATP synthase subunit α [*Glycine max*]	ATP hydrolysis coupled proton transport	55.3/52.6	6.23/6.4	721	20	33	+111.2	+54.1
11	gi|543866	ATP synthase subunit α [*Pisum sativum*]	ATP hydrolysis coupled proton transport	55/49.1	6.01/6.4	426	12	20	+29.8	+22.6
12	gi|55584146	NADH-ubiquinone oxidoreductase 24 kDa subunit [*Arabidopsis thaliana*]	Mitochondrial electron transport, NADH to ubiquinone, response to oxidative stress	28.4/25.7	8.09/6.3	297	6	13	+30.0	+54.5
13	gi|118573090	Dihydrolipoyllysine-residue acetyltransferase component 2 of pyruvate dehydrogenase complex [*Arabidopsis thaliana*]	Pyruvate metabolic process, glycolysis	58.4/175	7.55/5.4	105	3	5	+131.2	+51.5
14	gi|255635235	Uknown protein [*Glycine max*]	Lipid metabolic process	39.8/37	4.64/4.7	55	1	2	+102.3	+179.6
15	gi|585272	Heat shock protein 70 kDa [*Pisum sativum*]	Protein folding, response to stress	72.3/71	5.81/5.8	595	12	14	−42.6	−60.6
16	gi|123650	Heat shock protein 70 kDa [*Petunia hybrida*]	Response to stress	71.2/72.8	5.1/5.3	1,996	41	36	−70.7	−50.0
17	gi|461735	Chaperonin CPN60-1 [*Cucurbita maxima*]	Response to stress, protein folding	61/32	5.63/6.7	193	3	6	−64.6	−64.5
18	gi|461736	Chaperonin CPN60-2. [*Cucurbita maxima*]	Response to stress, protein folding	61.1/59.9	6.28/5.4	1,159	26	35	−80.8	−74.0
19	gi|75100413	Isocitrate dehydrogenase [NAD] regulatory subunit 1 [*Arabidopsis thaliana*]	Isocitrate metabolic process, tricarboxylic acid cycle	39.9/40.9	7.08/5.85	86	1	2	−49.7	−60.0
20	gi|122064254	Isocitrate dehydrogenase [NAD] regulatory subunit 2 [*Arabidopsis thaliana*]	Tricarboxylic acid cycle	39.6/39.8	6.14/6	142	4	12	−50.4	−46.4
21	gi|255645357	Unknown protein [*Glycine max*]	Cell redox homeostasis, glycerol ether metabolic process	40.5/42	5.46/6.0	341	9	20	−37.6	−56.9

Proteins were isolated from mitochondria of lupine embryo axes grown for 12 h on Heller medium with 250 and 500 mM NaCl or without NaCl. Proteins were separated by 2D-IEF-PAGE electrophoresis using linear pH gradient 4–7

^a^Protein spot number according to Image Master Platinum software

^b^Accession number in NCBInr database

^c^Proteins homologue with the best parameters of identification

^d^Gene ontology annotations according to REVIGO

^e^Theoretical (theor) protein mass (kDa) and experimental (exp) protein mass

^f^Theoretical (theor) pI and experimental (exp) pI

^g^MASCOT Score is value which determines a probability-based Ion Score for each peptide mtch, which indicates the statistical significance of that MS/MS spectral assignment; this value is calculated by the formula *S* = −10log(*P*), where *P* is the probability that obtained result is random

^h^Number of peptide ions detected in identified protein

^i^Percentage of sequence coverage by matched peptides

^j,k^Percentage change of identified protein abundance under 250 and 500 mN NaCl treatment, respectively, compared to control

## Discussion

In this work, changes in mitochondria proteins composition under salinity condition are discussed. Twenty-one proteins showing significant changes in abundance under both 250 and 500 mM NaCl treatment were identified (Table 2). They were both up- and down-regulated in response to salinity treatment. There are only a few published data which concern the mitochondrial proteome response during salinity stress. Mitochondria proteome responses were analyzed in *Oryza sativa* roots during salt-induced PCD (Chen et al. 2009b) and in wheat under salt treatment (Jacoby et al. 2010). Results presented in this paper provide some new information about participation of mitochondrial proteins in response to salinity. Identified proteins are involved in mitochondria biogenesis, response to stress and different metabolic processes. The possible role of all identified proteins in response to salinity stress and salinity-induced PCD is discussed.

### Mitochondria biogenesis

Among the group of up-regulated proteins, there was particularly one protein, which was over-accumulated. It was identified as mitochondrial import inner membrane translocase subunit Tim17/22, whose abundance was more than 600 % as high in mitochondria isolated from lupine embryo axes treated with 500 mM NaCl for 12 h as compared to control. Induction of different mitochondrial import inner membrane translocase subunit Tim17/Tim22/Tim23 family was observed upon antimycin A and rotenone treatment in *Arabidopsis* suspension cell. It was postulated that the induction of genes encoding components of the mitochondrial protein import apparatus may allow recovery from stress condition (Lister et al. 2004). Thus, the accumulation of Tim17/22 subunit may participate in modulation of mitochondrial proteins composition upon stress conditions. Other proteins like chaperonins CPN60, CPN60-1 and CPN60-2 which could be involved in proteins import into mitochondria were also identified. These proteins were both up- and down-regulated in mitochondria from salinity-stressed embryonic axes. Chaperonin proteins are implicated in protein import into mitochondria and macromolecular assembly and may facilitate the correct folding of imported proteins and also prevent misfolding and promote the refolding and proper assembly of unfolded polypeptides generated under stress conditions in the mitochondrial matrix (Prasad and Stewart 1992). Mitochondrial-processing peptidase, which is involved in proteins import, was also up-regulated in lupine embryo axes treated with 250 and 500 mM NaCl. This enzyme cleaves presequences (transit peptides) from mitochondrial protein precursors by release of N-terminal transit peptides from precursor proteins imported into the mitochondrion. In this study, alpha subunits of heterodimer (alpha and beta) were identified. Mitochondrial elongation factor Tu that participates in the process of mitochondria biogenesis was accumulated in lupine embryo axes in response to salinity. The accumulation of mitochondrial elongation factor Tu was also stated in salt-stressed *Solanum lycopersicum* (Chen et al. 2009a). This protein promotes the GTP-dependent binding of aminoacyl-tRNA to the A-site of ribosomes during protein biosynthesis. Changes in the level of mitochondrial elongation factor Tu accumulation were also observed in embryo axes during imbibition of *Glycine max* (Yin et al. 2009). It was postulated that environmental stresses can both inhibit and stimulate different protein import pathways in plant mitochondria. Operation of the import process is central to mitochondrial biogenesis both to repair damaged organelles and to increase mitochondrial bulk during regrowth. Ensuring the fidelity of the process during stress may be important to tolerance of environmental stresses such as drought and would assist in recovery of plant growth (Taylor et al. 2003). Increased accumulation of group of proteins, which are involved in mitochondria biogenesis during salinity stress in lupine embryo axes mitochondria, may implicate their important function in stress response and partitioning in protective mechanisms.

### Response to stress

In mitochondria from salinity-stressed embryo axes both increased and decreased accumulation of heat shock protein HSP-70 was stated. Both increased and decreased HSP-70 accumulation level was also observed in pea mitochondria during chilling, drought and paraquat-induced stress condition (Taylor et al. 2005). Accumulation of mitochondrial HSP-70 and the relation between HSP-70 and NaCl-induced PCD have been discussed (Chen et al. 2009b). It is suggested that mitochondrial HSP70 is a potential candidate for PCD regulation (Chen et al. 2009b). Accumulation of heat-shock proteins was reported for numerous species and was observed more frequently when NaCl was applied in high concentration, which could be discussed as salt shock response (Shavrukov 2013). Increased expression of HSP genes was observed within minutes after sudden exposure of rice to salinity (Zou et al. 2009). HSPs act as molecular chaperones in folding, assembling, and transporting of proteins. It is believed that the accumulations of HSPs play a pivotal role in abiotic stress responses in plants (Wang et al. 2004). The accumulation of mitochondrial Mn-SOD in lupine embryo axes in response to salinity stress was observed. Jacoby et al. (2010) also observed accumulation of Mn-SOD in mitochondria as a response of wheat to salt and discussed it as a key determination of whole plant salinity tolerance. Chen et al. (2009b) identified Cu/Zn-SOD as PCD-induced mitochondrial antioxidant enzyme. Mn-SOD activity is also essential for keeping the REDOX state of mitochondria and whole cell in balance (Giraud et al. 2012). In mitochondria isolated from embryonic axes cultured for 12 h in the presence of NaCl, the level of formic acid dehydrogenase increased. Formate dehydrogenase is a mitochondrial enzyme which occurs mainly in non-green tissues and participates in the oxidation of formate to CO_2_ in the presence of NAD^+^. Formate metabolisation by formate dehydrogenase produces NADH. In intact mitochondria, this NADH can be oxidized via the oxidative electron transport pathway. This electron transport is coupled to ATP synthesis (Oliver 1981). However, despite an accumulation of formate dehydrogenase under NaCl stress, the level of oxygen consumption by lupine mitochondria decreased (Table 1). Analyses of mitochondria isolated from plants subjected to saline conditions showed also inhibition of respiratory rate (Jacoby et al. 2011). Although no differences in ultrastructure of isolated mitochondria were observed, in situ ultrastructure analyses showed swollen cristae, narrowed mitochondrial intermembrane space, mitochondrial matrix with low electron density in axes grown on medium with 250 mM NaCl and morphological abnormalities with obscure cristae and vesicular-shaped inner membrane in axes treated with 500 mM NaCl (Wojtyla et al. 2013). The increase in the level of formate dehydrogenase was observed in response to various biotic and abiotic stresses (David et al. 2010). Accumulation of mitochondrial formate dehydrogenase precursor was observed in salt stress tomato seedlings (Chen et al. 2009a). According to result of AtGenExpress project (Kilian et al. 2007) visualized in *Arabidopsis* eFP Browser (Winter et al. 2007), the expression level of formate dehydrogenase increased in response to 150 mM NaCl. The exact physiological function of this enzyme in response to stresses has not been fully understood. The production of methanol, which can be converted to formate, may increase under stress conditions. Formate can arise from various pathways including photorespiration, cell wall degradation or synthesis, and glycolysis (Ambard-Bretteville et al. 2003; Hourton-Cabassa et al. 1998). In stressed plants, formate accumulation may result from enhancement of any of these pathways. In such cases, formate dehydrogenase might protect mitochondria against toxic effects of high concentration of formate (David et al. 2010). To better understand the role of formate dehydrogenase and its function in response to salinity stress, further analyses have to be done.

### Metabolic processes

In this study, decrease in accumulation of isocitrate dehydrogenase regulatory subunit 2 and 3 in lupine embryo axes was stated. Isocitrate dehydrogenase performs an important role in the oxidative function of the citric acid cycle and plays an essential role in defense against oxidative stress by supplying NADH for antioxidant systems. The suppression of its activity enhances the susceptibility of animal cells to apoptosis induction (Jung and Park 2011). Involvement of pyruvate dehydrogenase in plant response to abiotic stresses was reported for chilling, drought, and paraquat (Taylor et al. 2005), as well as for salt and sorbitol treatment (Rapala-Kozik et al. 2012). Accumulation of dihydrolipoyllysine-residue acetyltransferase component E2 of pyruvate dehydrogenase complex was observed in mitochondria from lupine embryo axes treated with 250 and 500 mM NaCl. Accumulation of alpha-subunit of ATP synthase increased in mitochondria from salinity-stressed lupine embryonic axes. Higher level of ATP synthase subunit alpha and beta was observed in pea during abiotic stresses (Taylor et al. 2005), whereas decrease in their abundance was stated under oxidative stress in *Arabidopsis* cells suspension culture (Sweetlove et al. 2002). The decrease of beta subunit of ATP synthase was reported under salt stress in tomato seedlings (Chen et al. 2009a) and during salt-induced PCD in rice (Chen et al. 2009b). Reduced accumulation of 24 kDa subunit of NADH-ubiquinone oxidoreductase was observed in salinity-stressed lupine embryo axes. Similar observations were made in oxidatively stressed *Arabidopsis* cells (Sweetlove et al. 2002). Modifications in proteins abundance and metabolic adjustment as a response to stress may play important role to retrieve homeostasis after salt shock.

## Conclusion

Our current study was aimed at characterizing the effect of salt shock onto mitochondria proteome. Using 2D-IEF-PAGE and LC–MS–MS/MS techniques, 21 proteins showing significant increase or decrease after 12 h of salinity treatment were successfully identified. This study indicates that the identified proteins comprise three main functional categories and are essential for mitochondria biogenesis, stress response and metabolic processes. In addition to proteins, which were reported previously to participate in salinity stress response and/or salinity-induced PCD, significant increase in the level of Tim17/22 subunit was also found. This protein, to our knowledge, has not been previously reported in the salinity response. Although accumulation of this protein was shown in response to other than salinity stresses, its exact function in stress response is still not fully understood. Future works should focus on comparative proteomics approaches and advanced functional analysis of the differentially expressed proteins for better and comprehensive understanding of mechanisms of mitochondria response to salinity stress/shock, which at higher salt concentration may involve execution of cell death, with a view of using this knowledge in plant breeding for stress environments.

### **Author contribution**

Ł. Wojtyla cultured lupine embryo axes, obtained mitochondrial fraction and prepared protein extracts, conducted electrophoresis, studied oxygen consumption, analysed results obtained from digital comparison of electrophorograms and from MS/MS protein identification and drafted the manuscript. A. Kosmala performed digital analysis of 2D gels. M. Garnczarska designed and coordinated the study and was responsible for verification of the paper.

## Electronic supplementary material

Below is the link to the electronic supplementary material.

Electronic Supplementary Material 1

Data processed by Mascot Distiller followed by Mascot Search (Matrix Science, London, UK). For each identified protein all matched tryptic peptides sequences are listed.

Supplementary material 1 (PDF 781 kb)
