# Supplementary material for: Lupine embryo axes under salinity stress. II. Mitochondrial proteome response
Source: Acta Physiol Plant. 2013 Apr 3;35(8):2383–92. doi: 10.1007/s11738-013-1273-2 (PMC4372824; doi:10.1007/s11738-013-1273-2)
Supplement: Supplementary file 1 — Supplementary material 1 (PDF 781 kb) [file 11738_2013_1273_MOESM1_ESM.pdf]

Electronic Supplementary Material 1

Acta Physiologiae Plantarum

Lupine embryo axes under salinity stress. II. Mitochondrial proteome response.

Łukasz Wojtyla[1], Arkadiusz Kosmala[2], Małgorzata Garnczarska[1*]

[1]Department of Plant Physiology, Faculty of Biology, Adam Mickiewicz University, ul. Umultowska 89, 61-614 Poznań, Poland, [2]Laboratory of Cytogenetics, Institute of Plant Genetics, Polish Academy of Sciences, ul. Strzeszyńska 34, 60-479 Poznań, Poland,

*Corresponding author: M. Garnczarska, tel. +48 618295894 fax. +48 618295887, e-mail: garnczar@amu.edu.pl

Data processed by Mascot Distiller followed by Mascot Search (Matrix Science, London, UK). For each identified protein all matched tryptic peptides sequences are listed. Protein's spots no. correspond to no. in figure 2. Gi number – index of protein in NCBInr database, mass – molecular mass of protein, score – Mascot score result, queries matched – number of tryptic peptide match in sequence of protein, emPAI – the exponentially modified protein abundance index, query – index of tryptic peptide, observed – observed molecular mass of protein, Mr(expt) and Mr(calc) – predicted molecular mass of protein, ppm – fraction expressed as parts per million, miss – number of missed trypsin cleavage sites, score – ion Mascot scores, expect – frequency this match would occur by chance, rank - rank of peptide match, peptide – the peptide string that was matched.

5961

gi|118573090 **Mass:** 58431 **Score:** 105 **Queries matched:** 3 **emPAI:** 0.17

Dihydrolipoyllysine-residue acetyltransferase component 2 of pyruvate dehydrogenase complex [Arabidopsis thaliana]

Query	Observed	Mr(expt)	Mr(calc)	ppm	Miss	Score	Expect	Rank	Peptide
**39**	343.2224	684.4302	684.4282	2.90	0	31	0.0059	1	K.AAALALR.K

**308**	500.8068	999.5990	999.5964	2.55	0	59	2e-06	1	R.ISVNDLVIK.A
**523**	636.3550	1270.6955	1270.6921	2.66	0	51	3e-05	1	R.VIDGAIGAEWLK.A

6200

gi|55584146 **Mass:** 28370 **Score:** 297 **Queries matched:** 6 **emPAI:** 0.87

NADH-ubiquinone oxidoreductase 24 kDa subunit [Arabidopsis thaliana]

**Query**	**Observed**	**Mr(expt)**	**Mr(calc)**	**ppm**	**Miss**	**Score**	**Expect**	**Rank**	**Peptide**
**120**	408.2415	814.4684	814.4800	-14.3	0	31	0.011	1	K.VVEIVEK.L
**686**	542.9269	1625.7589	1625.7548	2.54	0	(59)	7.3e-06	1	R.VYEVATFYSMFNR.A
**687**	813.8879	1625.7613	1625.7548	3.97	0	100	5.3e-10	1	R.VYEVATFYSMFNR.A
**692**	548.2583	1641.7530	1641.7497	2.02	0	(74)	4.8e-07	1	R.VYEVATFYSMFNR.A + Oxidation (M)
**693**	821.8846	1641.7547	1641.7497	3.06	0	(100)	1.2e-09	1	R.VYEVATFYSMFNR.A + Oxidation (M)
**730**	579.6159	1735.8259	1735.8208	2.91	0	38	0.001	1	K.YHLLVCGTTPCMIR.G + 2 Carbamidomethyl (C); Oxidation (M)

6052

gi|231585 **Mass:** 55296 **Score:** 721 **Queries matched:** 20 **emPAI:** 1.97

ATP synthase subunit alpha [Glycine max]

**Query**	**Observed**	**Mr(expt)**	**Mr(calc)**	**ppm**	**Miss**	**Score**	**Expect**	**Rank**	**Peptide**
**56**	351.7237	701.4328	701.4323	0.74	0	37	0.0014	1	R.LTEVLK.Q
**65**	360.1886	718.3626	718.3610	2.26	0	32	0.0087	1	K.GGLTSER.K
**82**	378.2244	754.4343	754.4337	0.84	0	49	3.4e-05	1	K.APGIIER.K
**88**	384.6896	767.3646	767.3636	1.34	0	37	0.0013	1	-.MEFSVR.A
**91**	387.2298	772.4449	772.4443	0.85	0	49	0.00022	1	R.VGSAAQLK.A
**119**	408.2351	814.4556	814.4548	0.93	0	39	0.0019	1	R.ELIIGDR.Q
**160**	430.7558	859.4970	859.4949	2.37	0	(38)	0.0019	1	R.QMSLLLR.R
**179**	438.7536	875.4926	875.4899	3.19	0	49	0.0001	1	R.QMSLLLR.R +

							7		Oxidation (M)
**183**	442.7170	883.4195	883.4148	5.33	0	54	2.5e-05	1	R.GALSDHER.R
**272**	486.7776	971.5407	971.5400	0.74	0	85	3.3e-08	1	R.VVSVGDGIAR.V
**344**	513.8008	1025.5870	1025.5869	0.10	0	62	1.6e-06	1	K.AVDSLVPIGR.G
**359**	522.2904	1042.5662	1042.5659	0.27	0	83	6e-08	1	R.TGSIVDVPAGK.A
**511**	602.3338	1202.6530	1202.6506	1.96	0	77	1.4e-07	1	R.AAELTTLLESR.I
**517**	605.8447	1209.6748	1209.6718	2.51	0	67	8e-07	1	R.VVDALGVPIDGR.G
**526**	613.8146	1225.6147	1225.6125	1.77	0	49	8.2e-05	1	K.SVHEPMQTGLK.A
**571**	642.3534	1282.6922	1282.6921	0.05	0	64	1.8e-06	1	K.QPQYAPLPIEK.Q
**586**	434.2541	1299.7404	1299.7398	0.48	0	(40)	0.00037	1	K.TAIAIDTILNQK.Q
**587**	650.8781	1299.7417	1299.7398	1.49	0	106	8.8e-11	1	K.TAIAIDTILNQK.Q
**678**	480.2879	1437.8419	1437.8416	0.24	0	53	6.9e-06	1	R.GIRPAINVGLSVSR.V
**742**	513.2531	1536.7374	1536.7361	0.81	0	37	0.0014	1	R.EAFPGDVFYLHSR.L

6072

gi|543866 **Mass:** 55011 **Score:** 426 **Queries matched:** 12 **emPAI:** 0.93

ATP synthase subunit alpha [Pisum sativum]

Query	Observed	Mr(expt)	Mr(calc)	ppm	Miss	Score	Expect	Rank	Peptide
**58**	351.7239	701.4332	701.4323	1.20	0	31	0.0064	1	R.LTEVLK.Q
**83**	378.2253	754.4361	754.4337	3.20	0	44	0.00012	1	K.APGIIER.K
**95**	384.6893	767.3641	767.3636	0.69	0	38	0.00096	1	-.MEFSVR.A
**102**	390.2069	778.3993	778.3973	2.52	0	39	0.0013	1	K.IAQYER.D
**132**	408.2347	814.4548	814.4548	-0.05	0	45	0.00041	1	R.ELIIGDR.Q
**169**	430.7561	859.4976	859.4949	3.11	0	31	0.0092	1	R.QMSLLLR.R
**181**	442.7172	883.4198	883.4148	5.65	0	57	1.3e-05	1	R.GALSDHER.R
**271**	486.7786	971.5427	971.5400	2.78	0	76	2.4e-07	1	R.VVSVGDGIAR.V
**348**	522.2911	1042.5676	1042.5659	1.65	0	62	7.6e-06	1	R.TGSIVDVPAGK.A
**519**	602.3343	1202.6540	1202.6506	2.77	0	107	1.1e-10	1	R.AAELTTLLESR.I
**525**	605.8453	1209.6760	1209.6718	3.52	0	65	9.7e-07	1	R.VVDALGVPIDGR.G
**579**	642.3552	1282.6958	1282.6921	2.87	0	57	8.8e-	1	K.QPQYAPLPIEK.Q

							06		

6085

gi|29839695 **Mass:** 54368 **Score:** 259 **Queries matched:** 6 **emPAI:** 0.32

Probable mitochondrial-processing peptidase subunit alpha-1 [Arabidopsis thaliana]

Query	Observed	Mr(expt)	Mr(calc)	ppm	Miss	Score	Expect	Rank	Peptide
**309**	490.2640	978.5134	978.5134	0.00	0	35	0.0025	1	R.QILTYGER.K
**458**	560.2957	1118.5769	1118.5754	1.36	0	72	5.8e-07	1	K.SAVLMNLESR.M
**469**	568.2933	1134.5720	1134.5703	1.49	0	(71)	7.1e-07	1	K.SAVLMNLESR.M + Oxidation (M)
**811**	866.4235	1730.8324	1730.8264	3.50	0	104	3.5e-10	1	R.NPAFLDWEVNEELR.K
**812**	577.9517	1730.8333	1730.8264	4.02	0	(49)	0.00011	1	R.NPAFLDWEVNEELR.K
**813**	577.9525	1730.8357	1730.8264	5.39	0	(36)	0.0024	1	R.NPAFLDWEVNEELR.K

6120

gi|25090254 **Mass:** 49379 **Score:** 157 **Queries matched:** 4 **emPAI:** 0.28

Elongation factor Tu [Arabidopsis thaliana]

Query	Observed	Mr(expt)	Mr(calc)	ppm	Miss	Score	Expect	Rank	Peptide
**137**	409.2443	816.4741	816.4705	4.35	0	69	6.4e-07	1	R.TVGAGVVSK.V
**230**	464.2625	926.5104	926.5073	3.36	0	36	0.00074	1	K.VELPENVK.M
**326**	511.2646	1020.5147	1020.5128	1.89	0	42	0.0019	1	K.AIAFDEIDK.A
**671**	730.9020	1459.7895	1459.7857	2.60	0	67	8.6e-07	1	R.QVGVPSLVCFLNK.V + Carbamidomethyl (C)

6263

gi|109909540 **Mass:** 41315 **Score:** 171 **Queries matched:** 4 **emPAI:** 0.34

Formate dehydrogenase 1 [Oryza sativa subsp. japonica]

Query	Observed	Mr(expt)	Mr(calc)	ppm	Miss	Score	Expect	Rank	Peptide
**127**	398.1992	794.3838	794.3810	3.52	0	31	0.0082	1	R.AYDLEGK.T

**260**	478.2872	954.5598	954.5611	-1.28	0	40	0.0002 9	1	K.GVIIVNNAR.G
**620**	662.3123	1322.6101	1322.6064	2.80	0	93	3.8e-09	1	K.YEEDLDAMLPK.C
**633**	670.3100	1338.6054	1338.6013	3.10	0	(66)	1.5e-06	1	K.YEEDLDAMLPK.C + Oxidation (M)

6219

gi|464775 **Mass:** 25823 **Score:** 271 **Queries matched:** 8 **emPAI:** 0.58

Superoxide dismutase [Mn] [Hevea brasiliensis]

Query	**Observed**	**Mr(expt)**	**Mr(calc)**	**ppm**	**Miss**	**Score**	**Expect**	**Rank**	**Peptide**
291	502.7797	1003.5449	1003.5451	-0.17	0	(37)	0.0011	1	K.NVRPDYLK.N
293	502.7803	1003.5459	1003.5451	0.89	0	45	0.0002 2	1	K.NVRPDYLK.N
582	709.8444	1417.6743	1417.6738	0.33	0	71	4.9e-07	1	K.HHQTYITNYNK.A
585	709.8453	1417.6760	1417.6738	1.51	0	(54)	2.9e-05	1	K.HHQTYITNYNK.A
586	473.5660	1417.6761	1417.6738	1.56	0	(45)	0.0001 9	1	K.HHQTYITNYNK.A
587	473.5661	1417.6766	1417.6738	1.92	0	(60)	6e-06	1	K.HHQTYITNYNK.A
718	814.4483	1626.8820	1626.8829	-0.51	0	56	8e-06	1	K.LVVETTANQDPLVTK.G
720	814.4516	1626.8887	1626.8829	3.58	0	(37)	0.0006 8	1	K.LVVETTANQDPLVTK.G

6026

gi|7330642 **Mass:** 73032 **Score:** 1695 **Queries matched:** 35 **emPAI:** 1.63

Heat shock 70 kDa protein [Solanum tuberosum]

**Query**	**Observed**	**Mr(expt)**	**Mr(calc)**	**ppm**	**Miss**	**Score**	**Expect**	**Rank**	**Peptide**
**10**	316.1753	630.3361	630.3337	3.85	0	34	0.0069	1	K.LDAANK.A
**51**	351.2012	700.3879	700.3868	1.61	0	44	0.0004 3	1	K.VLQGER.E
**91**	387.7217	773.4289	773.4283	0.79	0	31	0.0083	1	R.NTTIPTK.K
**121**	405.6696	809.3247	809.3225	2.67	0	44	0.0001 1	1	R.EMASDNK.L + Oxidation (M)
**268**	484.2872	966.5598	966.5611	-1.33	0	(55)	4e-06	1	K.HLNITLTR.S
**270**	484.2887	966.5628	966.5611	1.80	0	57	2.1e-06	1	K.HLNITLTR.S
**276**	487.7498	973.4851	973.4828	2.36	0	78	1.7e-	1	K.VIENSEGAR.T

							07		
**291**	492.7892	983.5638	983.5651	-1.39	0	56	9.9e-06	1	K.GELLVGTPAK.R
**292**	492.7901	983.5656	983.5651	0.46	0	(38)	0.00059	1	K.GELLVGTPAK.R
**294**	492.7942	983.5739	983.5651	8.96	0	(35)	0.0011	1	K.GELLVGTPAK.R
**457**	582.3258	1162.6371	1162.6346	2.10	0	90	3.4e-09	1	R.GIETLGGIFTR.L
**479**	596.3218	1190.6291	1190.6295	-0.40	0	57	1.5e-05	1	R.TTPSVVAFNQK.G
**523**	618.7836	1235.5526	1235.5517	0.70	0	(33)	0.0023	1	R.SSGGLSEDEIDK.M
**524**	618.7846	1235.5547	1235.5517	2.38	0	37	0.00091	1	R.SSGGLSEDEIDK.M
**648**	702.3645	1402.7144	1402.7126	1.30	0	(92)	3.8e-09	1	K.DVDEVLLVGGMTR.V
**649**	468.5791	1402.7154	1402.7126	1.94	0	(50)	5.6e-05	1	K.DVDEVLLVGGMTR.V
**650**	702.3651	1402.7156	1402.7126	2.15	0	(91)	3.8e-09	1	K.DVDEVLLVGGMTR.V
**651**	702.3657	1402.7168	1402.7126	2.99	0	105	1.5e-10	1	K.DVDEVLLVGGMTR.V
**652**	702.3665	1402.7185	1402.7126	4.18	0	(34)	0.0026	1	K.DVDEVLLVGGMTR.V
**657**	473.9103	1418.7091	1418.7076	1.12	0	(37)	0.0016	1	K.DVDEVLLVGG<u>M</u>TR.V + Oxidation (M)
**658**	710.3631	1418.7117	1418.7076	2.93	0	(81)	5e-08	1	K.DVDEVLLVGG<u>M</u>TR.V + Oxidation (M)
**757**	522.2764	1563.8074	1563.8045	1.85	0	(66)	1.3e-06	1	K.AVITVPAYFNDAQR.Q
**758**	782.9110	1563.8075	1563.8045	1.90	0	76	1.2e-07	1	K.AVITVPAYFNDAQR.Q
**759**	782.9133	1563.8120	1563.8045	4.80	0	(33)	0.0023	1	K.AVITVPAYFNDAQR.Q
**828**	598.6388	1792.8945	1792.8956	-0.56	0	(62)	3.6e-06	1	K.SQVFSTAADNQTQVGIK.V
**830**	897.4557	1792.8969	1792.8956	0.78	0	122	4.3e-12	1	K.SQVFSTAADNQTQVGIK.V
**831**	598.6399	1792.8978	1792.8956	1.26	0	(47)	0.00011	1	K.SQVFSTAADNQTQVGIK.V
**832**	598.6401	1792.8986	1792.8956	1.69	0	(60)	5.8e-06	1	K.SQVFSTAADNQTQVGIK.V
**833**	897.4569	1792.8993	1792.8956	2.10	0	(100)	5.7e-10	1	K.SQVFSTAADNQTQVGIK.V
**834**	897.4574	1792.9003	1792.8956	2.64	0	(108)	8.8e-11	1	K.SQVFSTAADNQTQVGIK.V
**835**	598.6420	1792.9041	1792.8956	4.74	0	(52)	3.4e-05	1	K.SQVFSTAADNQTQVGIK.V
**881**	647.0080	1938.0021	1937.9993	1.43	0	(73)	1.9e-07	1	K.GVNPDEAVAMGAALQGGILR.G
**882**	970.0103	1938.0059	1937.9993	3.44	0	145	9e-15	1	K.GVNPDEAVAMGAALQGGILR.G
**892**	652.3389	1953.9949	1953.9942	0.34	0	(97)	1.6e-	1	K.GVNPDEAVA<u>M</u>GAALQ

							09		GGILR.G + Oxidation (M)
**893**	978.0120	1954.0095	1953.9942	7.82	0	(135)	2.9e-13	1	K.GVNPDEAVAMGAALQGGILR.G + Oxidation (M)

6042

gi|12644189 **Mass:** 61242 **Score:** 3151 **Queries matched:** 74 **emPAI:** 3.55

Chaperonin CPN60 [Arabidopsis thaliana]

Query	Observed	Mr(expt)	Mr(calc)	ppm	Miss	Score	Expect	Rank	Peptide
**15**	324.6742	647.3339	647.3313	4.04	0	37	0.0046	1	K.VTMGPK.G + Oxidation (M)
**26**	339.6818	677.3490	677.3497	-1.04	0	44	0.00058	1	K.FGVEAR.A
**27**	339.6826	677.3507	677.3497	1.50	0	(32)	0.0079	1	K.FGVEAR.A
**38**	345.1951	688.3757	688.3756	0.24	0	50	0.00015	1	K.DGVTVAK.S
**39**	345.1953	688.3760	688.3756	0.67	0	(36)	0.0038	1	K.DGVTVAK.S
**85**	393.2597	784.5049	784.5058	-1.15	0	53	1.2e-05	1	K.VLELALK.R
**86**	393.2604	784.5062	784.5058	0.48	0	(37)	0.00053	1	K.VLELALK.R
**114**	413.7555	825.4964	825.4960	0.46	0	73	2.7e-07	1	K.AGIIDPLK.V
**129**	422.2675	842.5204	842.5226	-2.52	0	(46)	0.00012	1	K.LSGGVAVLK.I
**130**	422.2684	842.5222	842.5226	-0.36	0	(52)	3e-05	1	K.LSGGVAVLK.I
**131**	422.2687	842.5229	842.5226	0.45	0	(36)	0.0012	1	K.LSGGVAVLK.I
**132**	422.2695	842.5245	842.5226	2.35	0	(41)	0.00036	1	K.LSGGVAVLK.I
**133**	422.2701	842.5255	842.5226	3.56	0	55	1.5e-05	1	K.LSGGVAVLK.I
**134**	424.2060	846.3974	846.3984	-1.20	0	45	0.00031	1	K.APGFGENR.K
**166**	436.7575	871.5004	871.5014	-1.20	0	(31)	0.0055	1	R.EIGELIAK.A
**168**	436.7586	871.5027	871.5014	1.40	0	(33)	0.0034	1	R.EIGELIAK.A
**169**	436.7590	871.5034	871.5014	2.25	0	42	0.00047	1	R.EIGELIAK.A
**211**	466.7555	931.4964	931.4975	-1.12	0	(62)	7.3e-06	1	R.VTDALNATK.A
**212**	466.7564	931.4983	931.4975	0.94	0	66	3.4e-06	1	R.VTDALNATK.A
**214**	466.7570	931.4994	931.4975	2.14	0	(51)	8.4e-05	1	R.VTDALNATK.A
**227**	470.7260	939.4375	939.4372	0.32	0	53	3e-05	1	K.GEYVDMVK.A
**244**	478.7219	955.4291	955.4321	-3.07	0	(40)	0.0004	1	K.GEYVDMVK.A +

							7		Oxidation (M)
**305**	508.7659	1015.5173	1015.5186	-1.24	0	70	8.4e-07	1	K.GVEDLADAVK.V
**306**	508.7664	1015.5182	1015.5186	-0.41	0	(62)	4.8e-06	1	K.GVEDLADAVK.V
**307**	508.7678	1015.5211	1015.5186	2.48	0	(63)	4.2e-06	1	K.GVEDLADAVK.V
**308**	508.7680	1015.5214	1015.5186	2.76	0	(42)	0.0005	1	K.GVEDLADAVK.V
**309**	508.7687	1015.5229	1015.5186	4.28	0	(38)	0.0012	1	K.GVEDLADAVK.V
**342**	530.2876	1058.5606	1058.5608	-0.14	0	65	7.5e-06	1	K.EGVITIQDGK.T
**343**	530.2881	1058.5616	1058.5608	0.79	0	(40)	0.0024	1	K.EGVITIQDGK.T
**409**	573.7846	1145.5546	1145.5564	-1.54	0	(85)	2.1e-08	1	K.IGGASEAEVGEK.K
**410**	573.7851	1145.5556	1145.5564	-0.67	0	(42)	0.00044	1	K.IGGASEAEVGEK.K
**411**	573.7853	1145.5561	1145.5564	-0.25	0	(59)	8.4e-06	1	K.IGGASEAEVGEK.K
**412**	573.7855	1145.5564	1145.5564	-0.02	0	(55)	2e-05	1	K.IGGASEAEVGEK.K
**413**	573.7855	1145.5565	1145.5564	0.12	0	(67)	1.3e-06	1	K.IGGASEAEVGEK.K
**414**	573.7857	1145.5568	1145.5564	0.36	0	92	4.8e-09	1	K.IGGASEAEVGEK.K
**415**	573.7870	1145.5594	1145.5564	2.62	0	(74)	2.9e-07	1	K.IGGASEAEVGEK.K
**416**	573.7870	1145.5594	1145.5564	2.62	0	(82)	4.6e-08	1	K.IGGASEAEVGEK.K
**417**	573.7871	1145.5596	1145.5564	2.81	0	(82)	4e-08	1	K.IGGASEAEVGEK.K
**418**	573.7879	1145.5613	1145.5564	4.27	0	(92)	6.9e-09	1	K.IGGASEAEVGEK.K
**458**	598.8717	1195.7289	1195.7288	0.04	0	(80)	1e-08	1	K.IGVQIIQNALK.T
**459**	598.8727	1195.7309	1195.7288	1.77	0	88	1.7e-09	1	K.IGVQIIQNALK.T
**460**	598.8731	1195.7315	1195.7288	2.27	0	(38)	0.00017	1	K.IGVQIIQNALK.T
**477**	609.7940	1217.5735	1217.5776	-3.37	0	83	3.1e-08	1	K.DDTVILDGAGDK.K
**501**	618.2973	1234.5800	1234.5798	0.17	0	(102)	4.6e-10	1	K.SVAAGMNAMDLR.R
**513**	626.2944	1250.5743	1250.5747	-0.33	0	104	2.9e-10	1	K.SVAAGMNAMDLR.R + Oxidation (M)
**514**	626.2966	1250.5786	1250.5747	3.09	0	(54)	2.8e-05	1	K.SVAAGMNAMDLR.R + Oxidation (M)
**525**	634.2927	1266.5709	1266.5696	1.02	0	(99)	7.9e-10	1	K.SVAAGMNAMDLR.R + 2 Oxidation (M)
**573**	443.2375	1326.6906	1326.6932	-1.94	0	(31)	0.0038	1	R.NVVIEQSWGAPK.V
**574**	664.3535	1326.6925	1326.6932	-0.53	0	(42)	0.00035	1	R.NVVIEQSWGAPK.V
**575**	664.3538	1326.6930	1326.6932	-0.11	0	(80)	4.7e-08	1	R.NVVIEQSWGAPK.V

**576**	664.3539	1326.6932	1326.6932	0.01	0	(64)	2e-06	1	R.NVVIEQSWGAPK.V
**577**	664.3542	1326.6938	1326.6932	0.48	0	(64)	2e-06	1	R.NVVIEQSWGAPK.V
**578**	664.3549	1326.6951	1326.6932	1.47	0	92	2.6e-09	1	R.NVVIEQSWGAPK.V
**579**	664.3563	1326.6980	1326.6932	3.60	0	(48)	7.3e-05	1	R.NVVIEQSWGAPK.V
**767**	797.9085	1593.8025	1593.8072	-2.98	0	(68)	1.1e-06	1	K.CELDDPLILIHEK.K + Carbamidomethyl (C)
**768**	797.9110	1593.8074	1593.8072	0.14	0	90	5.6e-09	1	K.CELDDPLILIHEK.K + Carbamidomethyl (C)
**769**	532.2766	1593.8078	1593.8072	0.38	0	(69)	6.1e-07	1	K.CELDDPLILIHEK.K + Carbamidomethyl (C)
**814**	831.4049	1660.7953	1660.7944	0.57	0	82	3.3e-08	1	K.LLEQDNPDLGYDAAK.G
**1019**	698.3423	2092.0050	2092.0106	-2.68	0	(54)	2.3e-05	1	R.MISTSEEIAQVGTISANGER.E
**1020**	698.3431	2092.0074	2092.0106	-1.53	0	(62)	3e-06	1	R.MISTSEEIAQVGTISANGER.E
**1021**	698.3437	2092.0094	2092.0106	-0.60	0	(75)	1.5e-07	1	R.MISTSEEIAQVGTISANGER.E
**1022**	698.3447	2092.0123	2092.0106	0.79	0	(76)	1.3e-07	1	R.MISTSEEIAQVGTISANGER.E
**1023**	698.3448	2092.0127	2092.0106	0.99	0	(65)	1.6e-06	1	R.MISTSEEIAQVGTISANGER.E
**1024**	698.3456	2092.0150	2092.0106	2.08	0	(38)	0.00071	1	R.MISTSEEIAQVGTISANGER.E
**1025**	698.3462	2092.0168	2092.0106	2.96	0	(61)	3.8e-06	1	R.MISTSEEIAQVGTISANGER.E
**1037**	703.6744	2108.0013	2108.0055	-2.03	0	(59)	6.5e-06	1	R.MISTSEEIAQVGTISANGER.E + Oxidation (M)
**1038**	703.6768	2108.0086	2108.0055	1.44	0	(85)	1.5e-08	1	R.MISTSEEIAQVGTISANGER.E + Oxidation (M)
**1039**	703.6774	2108.0102	2108.0055	2.22	0	(49)	6.7e-05	1	R.MISTSEEIAQVGTISANGER.E + Oxidation (M)
**1040**	1055.0188	2108.0230	2108.0055	8.31	0	86	1.3e-08	1	R.MISTSEEIAQVGTISANGER.E + Oxidation (M)
**1084**	745.6925	2234.0557	2234.0598	-1.80	0	(38)	0.00041	1	K.QVANATNDVAGDGTTCATVLTR.A + Carbamidomethyl (C)
**1086**	745.6941	2234.0605	2234.0598	0.35	0	(35)	0.00099	1	K.QVANATNDVAGDGTTCATVLTR.A + Carbamidomethyl (C)
**1087**	1118.0380	2234.0614	2234.0598	0.76	0	155	1.1e-15	1	K.QVANATNDVAGDGTTCATVLTR.A + Carbamidomethyl (C)
**1088**	745.6950	2234.0631	2234.0598	1.48	0	(56)	7.6e-06	1	K.QVANATNDVAGDGTTCATVLTR.A + Carbamidomethyl (C)
**1089**	1118.0392	2234.0638	2234.0598	1.83	0	(94)	1.4e-09	1	K.QVANATNDVAGDGTTCATVLTR.A + Carbamidomethyl (C)

6045

gi|2506275 **Mass:** 61173 **Score:** 1309 **Queries matched:** 28 **emPAI:** 1.68

Chaperonin CPN60-1 [Zea mays]

Query	Observed	Mr(expt)	Mr(calc)	ppm	Miss	Score	Expect	Rank	Peptide
**41**	345.1960	688.3775	688.3756	2.85	0	38	0.0031	1	K.DGVTVAK.S
**141**	422.2710	842.5274	842.5226	5.81	0	45	0.0001 4	1	K.LSGGVAVLK.I
**145**	424.2061	846.3975	846.3984	-1.01	0	40	0.001	1	K.APGFGENR.K
**168**	436.7580	871.5014	871.5014	-0.07	0	38	0.0011	1	R.EIGELIAK.A
**225**	466.7566	931.4986	931.4975	1.26	0	59	1.3e-05	1	R.VTDALNATK.A
**237**	470.7272	939.4398	939.4372	2.86	0	40	0.0005 9	1	K.GEYVDMVK.T
**333**	515.7753	1029.5360	1029.5342	1.75	0	73	4.1e-07	1	R.GVEELADAVK.V
**376**	532.7725	1063.5304	1063.5298	0.52	0	38	0.0017	1	K.LQTANFDQK.I
**445**	573.7852	1145.5558	1145.5564	-0.56	0	(58)	1.1e-05	1	K.IGGASEAEVGEK.K
**446**	573.7869	1145.5593	1145.5564	2.53	0	(58)	1.3e-05	1	K.IGGASEAEVGEK.K
**447**	573.7870	1145.5594	1145.5564	2.62	0	95	2.1e-09	1	K.IGGASEAEVGEK.K
**448**	573.7873	1145.5601	1145.5564	3.19	0	(92)	7e-09	1	K.IGGASEAEVGEK.K
**493**	598.8729	1195.7313	1195.7288	2.05	0	50	9.9e-06	1	K.IGVQIIQNALK.T
**524**	609.7970	1217.5795	1217.5776	1.55	0	79	7.4e-08	1	K.DDTVILDGAGDK.K
**557**	634.2944	1266.5743	1266.5696	3.68	0	98	8.7e-10	1	K.SVAAGMNAMDLR.R + 2 Oxidation (M)
**570**	644.8497	1287.6849	1287.6823	2.04	0	62	3.1e-06	1	R.NVVIEQSFGAPK.V
**595**	665.2961	1328.5777	1328.5732	3.42	0	77	7.8e-08	1	R.SAIENSTSDYDK.E
**802**	634.3523	1900.0350	1900.0306	2.31	0	105	7e-11	1	K.AAVEEGIVPGGGVALL YASK.E
**805**	636.0176	1905.0311	1905.0320	- 0.48	0	(73)	7.3e-08	1	K.TPVHTIASNAGVEGAV VVGK.L
**806**	636.0193	1905.0361	1905.0320	2.17	0	(71)	1.2e-07	1	K.TPVHTIASNAGVEGAV VVGK.L
**807**	953.5263	1905.0381	1905.0320	3.19	0	110	1.6e-11	1	K.TPVHTIASNAGVEGAV VVGK.L
**808**	636.0201	1905.0385	1905.0320	3.41	0	(66)	3.7e-07	1	K.TPVHTIASNAGVEGAV VVGK.L
**809**	636.0204	1905.0395	1905.0320	3.95	0	(31)	0.0011	1	K.TPVHTIASNAGVEGAV

									VVGK.L
**883**	1047.0113	2092.0080	2092.0106	-1.23	0	(57)	1e-05	1	R.MISTSEEIAQVGTISANGER.E
**884**	698.3442	2092.0108	2092.0106	0.09	0	(68)	7.7e-07	1	R.MISTSEEIAQVGTISANGER.E
**885**	698.3443	2092.0110	2092.0106	0.19	0	(63)	2.9e-06	1	R.MISTSEEIAQVGTISANGER.E
**895**	703.6741	2108.0004	2108.0055	-2.46	0	(53)	2.5e-05	1	R.MISTSEEIAQVGTISANGER.E + Oxidation (M)
**896**	703.6754	2108.0044	2108.0055	-0.53	0	73	2.7e-07	1	R.MISTSEEIAQVGTISANGER.E + Oxidation (M)

6044

gi|2493646 **Mass:** 60897 **Score:** 2254 **Queries matched:** 46 **emPAI:** 2.80

Chaperonin CPN60-2 [Zea mays]

Query	Observed	Mr(expt)	Mr(calc)	ppm	Miss	Score	Expect	Rank	Peptide
**30**	324.6739	647.3333	647.3313	3.24	0	33	0.012	1	K.VTMGPK.G + Oxidation (M)
**42**	339.6820	677.3494	677.3497	-0.45	0	44	0.00061	1	K.FGVEAR.A
**44**	339.6824	677.3502	677.3497	0.76	0	(39)	0.002	1	K.FGVEAR.A
**54**	345.1961	688.3777	688.3756	3.06	0	42	0.0011	1	K.DGVTVAK.S
**142**	413.7554	825.4961	825.4960	0.20	0	53	2.3e-05	1	K.AGIIDPLK.V
**161**	422.2681	842.5216	842.5226	-1.10	0	59	6e-06	1	K.LSGGVAVLK.I
**166**	424.2063	846.3980	846.3984	-0.50	0	45	0.00026	1	K.APGFGENR.K
**169**	424.2085	846.4024	846.3984	4.70	0	(34)	0.0034	1	K.APGFGENR.K
**194**	436.7578	871.5011	871.5014	-0.42	0	53	3.7e-05	1	R.EIGELIAK.A
**234**	463.2256	924.4367	924.4375	-0.86	0	51	6e-05	1	K.AIFTEGCK.S + Carbamidomethyl (C)
**241**	466.7554	931.4962	931.4975	-1.31	0	66	3.4e-06	1	R.VTDALNATK.A
**368**	515.7741	1029.5336	1029.5342	-0.58	0	78	1.5e-07	1	R.GVEELADAVK.V
**369**	515.7753	1029.5359	1029.5342	1.67	0	(31)	0.0064	1	R.GVEELADAVK.V
**397**	532.7713	1063.5280	1063.5298	-1.68	0	43	0.00066	1	K.LQTANFDQK.I
**399**	532.7734	1063.5323	1063.5298	2.34	0	(30)	0.012	1	K.LQTANFDQK.I
**469**	573.7849	1145.5553	1145.5564	-1.00	0	(59)	8.1e-06	1	K.IGGASEAEVGEK.K
**470**	573.7861	1145.5576	1145.5564	1.03	0	(85)	2.1e-08	1	K.IGGASEAEVGEK.K

**472**	573.7866	1145.5586	1145.5564	1.92	0	(63)	3.2e-06	1	K.IGGASEAEVGEK.K
**473**	573.7869	1145.5593	1145.5564	2.53	0	(75)	2e-07	1	K.IGGASEAEVGEK.K
**474**	573.7872	1145.5598	1145.5564	2.93	0	95	2e-09	1	K.IGGASEAEVGEK.K
**475**	573.7878	1145.5611	1145.5564	4.12	0	(95)	2.9e-09	1	K.IGGASEAEVGEK.K
**516**	598.8709	1195.7272	1195.7288	-1.36	0	(54)	3.9e-06	1	K.IGVQIIQNALK.T
**517**	598.8719	1195.7293	1195.7288	0.38	0	83	5.6e-09	1	K.IGVQIIQNALK.T
**537**	609.7947	1217.5748	1217.5776	-2.27	0	(47)	0.00012	1	K.DDTVILDGAGDK.K
**538**	609.7957	1217.5769	1217.5776	-0.57	0	109	6.9e-11	1	K.DDTVILDGAGDK.K
**558**	618.2976	1234.5807	1234.5798	0.70	0	99	9.9e-10	1	K.SVAAGMNAMDLR.R
**559**	618.2997	1234.5848	1234.5798	4.05	0	(37)	0.0013	1	K.SVAAGMNAMDLR.R
**572**	626.2948	1250.5750	1250.5747	0.24	0	(80)	7.6e-08	1	K.SVAAGMNAMDLR.R + Oxidation (M)
**573**	626.2974	1250.5803	1250.5747	4.43	0	(36)	0.0018	1	K.SVAAGMNAMDLR.R + Oxidation (M)
**591**	634.2919	1266.5692	1266.5696	-0.33	0	(82)	3.9e-08	1	K.SVAAGMNAMDLR.R + 2 Oxidation (M)
**612**	644.8486	1287.6826	1287.6823	0.23	0	104	2.2e-10	1	R.NVVIEQSFGAPK.V
**613**	644.8492	1287.6838	1287.6823	1.19	0	(82)	3.3e-08	1	R.NVVIEQSFGAPK.V
**614**	644.8496	1287.6847	1287.6823	1.86	0	(81)	4.1e-08	1	R.NVVIEQSFGAPK.V
**634**	658.2865	1314.5584	1314.5575	0.69	0	59	4.5e-06	1	R.SAVENSTSDYDK.E
**930**	951.0239	1900.0332	1900.0306	1.37	0	100	2.6e-10	1	K.AAVEEGIVPGGGVALLYASK.E
**931**	634.3522	1900.0347	1900.0306	2.20	0	(92)	1.3e-09	1	K.AAVEEGIVPGGGVALLYASK.E
**936**	636.0170	1905.0293	1905.0320	-1.44	0	(63)	7.8e-07	1	K.TPVHTIASNAGVEGAVVGK.L
**937**	636.0176	1905.0309	1905.0320	-0.57	0	(71)	1e-07	1	K.TPVHTIASNAGVEGAVVGK.L
**938**	477.2663	1905.0361	1905.0320	2.15	0	(42)	9.1e-05	1	K.TPVHTIASNAGVEGAVVGK.L
**939**	636.0199	1905.0379	1905.0320	3.08	0	(52)	8.9e-06	1	K.TPVHTIASNAGVEGAVVGK.L
**940**	953.5270	1905.0395	1905.0320	3.95	0	93	7e-10	1	K.TPVHTIASNAGVEGAVVGK.L
**1009**	698.3437	2092.0092	2092.0106	-0.69	0	(94)	2.1e-09	1	R.MISTSEEIAQVGTISANGER.E
**1010**	1047.0127	2092.0108	2092.0106	0.11	0	131	4.4e-13	1	R.MISTSEEIAQVGTISANGER.E
**1011**	1047.0128	2092.0110	2092.0106	0.20	0	(85)	1.5e-08	1	R.MISTSEEIAQVGTISANGER.E

**1012**	1047.0157	2092.0168	2092.0106	2.98	0	(117)	9.5e-12	1	R.MISTSEEIAQVGTISANGER.E
**1025**	703.6745	2108.0016	2108.0055	-1.85	0	(71)	4.4e-07	1	R.MISTSEEIAQVGTISANGER.E + Oxidation (M)

6162

gi|255635235 **Mass:** 39774 **Score:** 55 **Queries matched:** 1 **emPAI:** 0.08

unknown [Glycine max]

Query	Observed	Mr(expt)	Mr(calc)	ppm	Miss	Score	Expect	Rank	Peptide
**454**	638.8357	1275.6569	1275.6571	-0.13	0	55	0.0008	1	K.INTANQIFNNK.L

6237

gi|87240460 **Mass:** 19294 **Score:** 566 **Queries matched:** 17 **emPAI:** 0.17

Mitochondrial import inner membrane translocase, subunit Tim17/22 [Medicago truncatula]

Query	Observed	Mr(expt)	Mr(calc)	ppm	Miss	Score	Expect	Rank	Peptide
**657**	550.3130	1098.6115	1098.6145	-2.73	0	(54)	0.0031	1	K.AAGIGAVQAVSR.E
**658**	550.3137	1098.6128	1098.6145	-1.58	0	(85)	2.6e-06	1	K.AAGIGAVQAVSR.E
**659**	550.3137	1098.6129	1098.6145	-1.49	0	(85)	2.6e-06	1	K.AAGIGAVQAVSR.E
**660**	550.3137	1098.6129	1098.6145	-1.47	0	(67)	0.00015	1	K.AAGIGAVQAVSR.E
**661**	550.3142	1098.6138	1098.6145	-0.69	0	(67)	0.00017	1	K.AAGIGAVQAVSR.E
**662**	550.3143	1098.6141	1098.6145	-0.44	0	(86)	2.4e-06	1	K.AAGIGAVQAVSR.E
**663**	550.3144	1098.6142	1098.6145	-0.35	0	(93)	4.8e-07	1	K.AAGIGAVQAVSR.E
**664**	550.3144	1098.6142	1098.6145	-0.31	0	107	1.6e-08	1	K.AAGIGAVQAVSR.E
**667**	550.3151	1098.6156	1098.6145	0.93	0	(49)	0.012	1	K.AAGIGAVQAVSR.E
**669**	550.3153	1098.6160	1098.6145	1.37	0	(71)	5.9e-05	1	K.AAGIGAVQAVSR.E
**670**	550.3153	1098.6160	1098.6145	1.37	0	(72)	5.6e-05	1	K.AAGIGAVQAVSR.E
**671**	550.3154	1098.6163	1098.6145	1.62	0	(59)	0.001	1	K.AAGIGAVQAVSR.E
**672**	550.3155	1098.6165	1098.6145	1.78	0	(65)	0.00026	1	K.AAGIGAVQAVSR.E
**673**	550.3156	1098.6166	1098.6145	1.88	0	(49)	0.011	1	K.AAGIGAVQAVSR.E

**674**	550.3156	1098.6167	1098.6145	1.98	0	(66)	0.00019	1	K.AAGIGAVQAVSR.E
**676**	550.3158	1098.6170	1098.6145	2.20	0	(89)	1e-06	1	K.AAGIGAVQAVSR.E
**677**	550.3168	1098.6190	1098.6145	4.08	0	(73)	3.7e-05	1	K.AAGIGAVQAVSR.E

6110

gi|75100413 **Mass:** 39602 **Score:** 86 **Queries matched:** 1 **emPAI:** 0.08

Isocitrate dehydrogenase [NAD] regulatory subunit 1 [Arabidopsis thaliana]

Query	Observed	Mr(expt)	Mr(calc)	ppm	Miss	Score	Expect	Rank	Peptide
**552**	640.8209	1279.6273	1279.6231	3.32	0	86	2e-08	1	K.LADGLFLESCR.E + Carbamidomethyl (C)

6107

gi|122064254 **Mass:** 39565 **Score:** 142 **Queries matched:** 4 **emPAI:** 0.35

Isocitrate dehydrogenase [NAD] regulatory subunit 2 [Arabidopsis thaliana]

Query	Observed	Mr(expt)	Mr(calc)	ppm	Miss	Score	Expect	Rank	Peptide
**471**	593.3412	1184.6678	1184.6652	2.18	0	42	0.00019	1	K.SLPEGLLESIK.K
**661**	712.3370	1422.6594	1422.6568	1.88	0	68	1.1e-06	1	K.YAFEYAYLNNR.K
**662**	475.2273	1422.6600	1422.6568	2.26	0	(32)	0.0038	1	K.YAFEYAYLNNR.K
**889**	829.0768	2484.2085	2484.2020	2.64	0	58	7.7e-06	1	R.ENTEGEYAGLEHEVVPGVVESLK.V

6023

gi|585272 **Mass:** 72257 **Score:** 595 **Queries matched:** 12 **emPAI:** 0.63

Heat shock 70 kDa protein [Pisum sativum]

Query	Observed	Mr(expt)	Mr(calc)	ppm	Miss	Score	Expect	Rank	Peptide
**40**	350.7222	699.4299	699.4279	2.87	0	46	0.00016	1	K.ALIDIR.N
**76**	380.7136	759.4127	759.4126	0.06	0	33	0.006	1	K.DANISIK.D
**261**	484.2877	966.5608	966.5611	-0.29	0	56	3.8e-	1	K.HLNITLTR.S

							06		
**268**	487.7419	973.4693	973.4828	-13.95	0	38	0.0011	1	K.VIENSEGAR.T
**406**	573.7870	1145.5594	1145.5564	2.63	0	49	8.2e-05	1	K.EIEDAVSDLR.T
**451**	596.3220	1190.6294	1190.6295	-0.15	0	64	3.5e-06	1	R.TTPSVVAFNQK.S
**634**	702.3660	1402.7175	1402.7126	3.51	0	96	1.3e-09	1	K.DVDEVLLVGGMTR.V
**828**	897.4529	1792.8912	1792.8956	-2.42	0	107	1.3e-10	1	K.SQVFSTAADNQTQVGIK.V
**829**	598.6387	1792.8942	1792.8956	-0.77	0	(53)	2.9e-05	1	K.SQVFSTAADNQTQVGIK.V
**830**	598.6400	1792.8981	1792.8956	1.39	0	(77)	1.4e-07	1	K.SQVFSTAADNQTQVGIK.V
**839**	602.9736	1805.8989	1805.8981	0.42	0	(70)	5.2e-07	1	R.IINEPTAAALSYGMNNK.E
**840**	903.9576	1805.9006	1805.8981	1.37	0	124	1.9e-12	1	R.IINEPTAAALSYGMNNK.E

6003

gi|123650 **Mass:** 71182 **Score:** 1996 **Queries matched:** 41 **emPAI:** 3.05

Heat shock cognate 70 kDa protein [Petunia hybrida]

Query	Observed	Mr(expt)	Mr(calc)	ppm	Miss	Score	Expect	Rank	Peptide
**43**	344.2054	686.3963	686.3963	0.08	0	41	0.0012	1	R.LIGDAAK.N
**103**	387.7216	773.4286	773.4283	0.38	0	33	0.0055	1	R.NTTIPTK.K
**121**	402.7268	803.4390	803.4389	0.17	0	38	0.0016	1	K.ITITNDK.G
**146**	417.7055	833.3965	833.3953	1.43	0	50	0.00013	1	R.MVQEAEK.Y
**197**	454.6966	907.3786	907.3779	0.77	0	32	0.0033	1	K.CMEPVEK.C + Carbamidomethyl (C); Oxidation (M)
**424**	592.3279	1182.6412	1182.6397	1.28	0	41	0.00054	2	K.FELSGIPPAPR.G
**450**	608.3288	1214.6429	1214.6441	-0.98	0	(98)	1.2e-09	1	K.DAGVIAGLNVMR.I
**451**	608.3307	1214.6468	1214.6441	2.18	0	(82)	5.1e-08	1	K.DAGVIAGLNVMR.I
**452**	608.3314	1214.6483	1214.6441	3.42	0	(46)	0.00017	1	K.DAGVIAGLNVMR.I
**467**	614.8175	1227.6204	1227.6207	-0.31	0	72	4e-07	1	R.VEIIANDQGNR.T
**468**	614.8187	1227.6228	1227.6207	1.68	0	(45)	0.00022	1	R.VEIIANDQGNR.T
**475**	616.3276	1230.6406	1230.6391	1.26	0	100	8e-10	1	K.DAGVIAGLNVMR.I +

									Oxidation (M)
**529**	426.8816	1277.6230	1277.6227	0.27	0	(32)	0.0067	1	R.MVNHFVQEFK.R
**530**	639.8190	1277.6234	1277.6227	0.58	0	61	7.8e-06	1	R.MVNHFVQEFK.R
**531**	426.8819	1277.6239	1277.6227	0.96	0	(38)	0.0018	1	R.MVNHFVQEFK.R
**546**	647.8168	1293.6191	1293.6176	1.17	0	(54)	3.6e-05	1	R.MVNHFVQEFK.R + Oxidation (M)
**573**	657.3144	1312.6142	1312.6122	1.58	0	(69)	9.7e-07	1	R.FEELNMDLFR.K
**574**	657.3145	1312.6144	1312.6122	1.75	0	(74)	3.3e-07	1	R.FEELNMDLFR.K
**592**	665.3108	1328.6070	1328.6071	-0.03	0	76	1.7e-07	1	R.FEELNMDLFR.K + Oxidation (M)
**620**	453.5434	1357.6083	1357.6084	-0.11	0	(47)	7.9e-05	1	K.NALENYAYNMR.N
**621**	679.8119	1357.6093	1357.6084	0.63	0	85	1.3e-08	1	K.NALENYAYNMR.N
**631**	458.8754	1373.6045	1373.6033	0.81	0	(32)	0.0035	1	K.NALENYAYNMR.N + Oxidation (M)
**632**	687.8099	1373.6052	1373.6033	1.38	0	(73)	3.2e-07	1	K.NALENYAYNMR.N + Oxidation (M)
**639**	693.8685	1385.7224	1385.7224	0.01	0	82	3.5e-08	1	K.ELESICNPIIAK.M + Carbamidomethyl (C)
**660**	471.5885	1411.7436	1411.7420	1.16	0	(33)	0.0025	1	K.SSVHDVVLVGGSTR.I
**663**	706.8798	1411.7451	1411.7420	2.21	0	74	1.8e-07	1	K.SSVHDVVLVGGSTR.I
**664**	471.5892	1411.7459	1411.7420	2.78	0	(41)	0.00032	1	K.SSVHDVVLVGGSTR.I
**676**	718.8782	1435.7419	1435.7460	-2.83	0	78	1.7e-07	1	K.VQQLLQDFFNGK.E
**677**	479.5903	1435.7492	1435.7460	2.22	0	(34)	0.0049	1	K.VQQLLQDFFNGK.E
**678**	718.8824	1435.7503	1435.7460	3.05	0	(76)	2.5e-07	1	K.VQQLLQDFFNGK.E
**698**	737.3468	1472.6790	1472.6784	0.47	0	87	1.2e-08	1	R.TTPSYVGFTDTER.L
**820**	553.9702	1658.8888	1658.8879	0.55	0	(75)	1.4e-07	1	R.IINEPTAAAIAYGLDK.K
**821**	830.4519	1658.8892	1658.8879	0.79	0	125	1.5e-12	1	R.IINEPTAAAIAYGLDK.K
**822**	830.4538	1658.8931	1658.8879	3.13	0	(117)	8.6e-12	1	R.IINEPTAAAIAYGLDK.K
**826**	831.4194	1660.8242	1660.8243	-0.07	0	(123)	3e-12	1	K.NQVAMNPINTVFDAK.R
**837**	838.3696	1674.7246	1674.7234	0.71	0	80	4e-08	1	K.ATAGDTHLGGEDFDNR.M
**840**	559.9471	1676.8196	1676.8192	0.23	0	(48)	0.00012	1	K.NQVAMNPINTVFDAK.R + Oxidation (M)
**841**	839.4174	1676.8202	1676.8192	0.59	0	142	4.5e-14	1	K.NQVAMNPINTVFDAK.R + Oxidation (M)
**842**	840.9216	1679.8286	1679.8267	1.09	0	96	1.6e-09	1	K.NAVVTVPAYFNDSQR.Q

**843**	560.9509	1679.8308	1679.8267	2.39	0	(71)	4.9e-07	1	K.NAVVTVPAYFNDSQR.Q
**1093**	858.7635	2573.2688	2573.2609	3.06	0	99	4.2e-10	1	K.SINPDEAVAYGAAVQAAILSGEGNEK.V

6189

gi|461735 **Mass:** 61017 **Score:** 193 **Queries matched:** 3 **emPAI:** 0.16

Chaperonin CPN60-1 [Cucurbita maxima]

Query	Observed	Mr(expt)	Mr(calc)	ppm	Miss	Score	Expect	Rank	Peptide
**716**	636.0188	1905.0345	1905.0320	1.30	0	55	4.1e-06	1	K.TPVHTIASNAGVEGAVVVGK.L
**765**	698.3451	2092.0135	2092.0106	1.38	0	(68)	8.6e-07	1	R.MISTSEEIAQVGTISANGER.E
**766**	1047.0141	2092.0136	2092.0106	1.45	0	105	1.6e-10	1	R.MISTSEEIAQVGTISANGER.E

6032

gi|461736 **Mass:** 61092 **Score:** 1159 **Queries matched:** 26 **emPAI:** 2.71

Chaperonin CPN60-2 [Cucurbita maxima]

Query	Observed	Mr(expt)	Mr(calc)	ppm	Miss	Score	Expect	Rank	Peptide
**26**	339.6820	677.3494	677.3497	-0.39	0	42	0.00098	1	K.FGVEAR.G
**34**	345.1955	688.3765	688.3756	1.37	0	36	0.004	1	K.DGVTVAK.S
**93**	393.2613	784.5079	784.5058	2.72	0	61	2.3e-06	1	K.VLELALK.R
**126**	413.7564	825.4982	825.4960	2.74	0	68	8.5e-07	1	K.AGIIDPLK.V
**148**	424.2073	846.4000	846.3984	1.89	0	42	0.00059	1	K.APGFGENR.K
**169**	436.7584	871.5021	871.5014	0.80	0	57	1.2e-05	1	R.EIGELIAK.A
**216**	463.2271	924.4397	924.4375	2.35	0	40	0.00081	1	R.AIFTEGCK.S + Carbamidomethyl (C)
**225**	466.7563	931.4981	931.4975	0.75	0	60	1.3e-05	1	R.VTDALNATK.A
**243**	477.7340	953.4534	953.4528	0.67	0	46	0.00019	1	K.GEYVDMIK.A
**263**	485.7338	969.4530	969.4477	5.41	0	(43)	0.00029	1	K.GEYVDMIK.A + Oxidation (M)

**320**	508.7677	1015.5209	1015.5186	2.27	0	70	8.2e-07	1	K.GVEDLADAVK.V
**323**	509.7746	1017.5346	1017.5342	0.40	0	60	9e-06	1	K.EGVITISDGK.T
**416**	573.7851	1145.5556	1145.5564	-0.67	0	85	2.2e-08	1	K.IGGASEAEVGEK.K
**418**	573.7871	1145.5596	1145.5564	2.77	0	(62)	4.7e-06	1	K.IGGASEAEVGEK.K
**455**	598.8726	1195.7307	1195.7288	1.55	0	68	1.7e-07	1	K.IGVQIIQNALK.T
**484**	618.2985	1234.5825	1234.5798	2.16	0	93	3.5e-09	1	K.SVAAGMNAMDLR.R
**499**	626.2956	1250.5767	1250.5747	1.56	0	(66)	1.7e-06	1	K.SVAAGMNAMDLR.R + Oxidation (M)
**508**	634.2927	1266.5708	1266.5696	0.92	0	(84)	2.2e-08	1	K.SVAAGMNAMDLR.R + 2 Oxidation (M)
**658**	715.8699	1429.7253	1429.7242	0.81	0	83	2.6e-08	1	R.GYISPYFITNQK.N
**659**	477.5828	1429.7265	1429.7242	1.67	0	(39)	0.00076	1	R.GYISPYFITNQK.N
**759**	532.2756	1593.8051	1593.8072	-1.34	0	67	1.2e-06	1	K.CELDDPLILIHEK.K + Carbamidomethyl (C)
**804**	831.4138	1660.8131	1660.7944	11.3	0	49	8.5e-05	1	K.LLEQDNPDLGYDAAK.G
**906**	951.0214	1900.0282	1900.0306	-1.26	0	97	4.2e-10	1	K.AAVEEGIVPGGGVALLYASK.E
**907**	634.3510	1900.0313	1900.0306	0.40	0	(83)	1.1e-08	1	K.AAVEEGIVPGGGVALLYASK.E
**976**	698.3445	2092.0116	2092.0106	0.47	0	(69)	6.9e-07	1	R.MISTSEEIAQVGTISANGER.E
**979**	703.6769	2108.0088	2108.0055	1.57	0	84	2.1e-08	1	R.MISTSEEIAQVGTISANGER.E + Oxidation (M)

6121

gi|255645357 **Mass:** 40466 **Score:** 341 **Queries matched:** 9 **emPAI:** 0.81

unknown [Glycine max]

Query	Observed	Mr(expt)	Mr(calc)	ppm	Miss	Score	Expect	Rank	Peptide
**86**	395.2224	788.4303	788.4280	3.00	0	53	0.0022	1	K.ADELTLK.K
**187**	448.1990	894.3833	894.3831	0.22	0	68	3e-05	1	K.AGEEYGGGR.D
**200**	454.7418	907.4690	907.4651	4.36	0	36	0.059	1	K.SLAPTYEK.V
**463**	563.7944	1125.5743	1125.5706	3.30	0	64	7.3e-05	1	K.YDVSGFPTLK.F
**671**	713.3519	1424.6892	1424.6824	4.79	0	74	7.2e-06	1	R.DLDDFVAFINEK.S
**739**	515.2681	1542.7824	1542.7777	3.04	0	(49)	0.002	1	K.LEEDVVIANLDADK.Y

**740**	772.3989	1542.7833	1542.7777	3.64	0	138	2.7e-12	1	K.LEEDVVIANLDADK.Y
**783**	548.2817	1641.8234	1641.8191	2.59	0	(42)	0.01	1	K.YGVSGYPTIQWFPK.G
**784**	821.9193	1641.8241	1641.8191	3.01	0	83	7e-07	1	K.YGVSGYPTIQWFPK.G
